# The effect of wafer thinning and thermal capacitance on chip temperature of SiC Schottky diodes during surge currents

**DOI:** 10.1038/s41598-023-46538-6

**Published:** 2023-11-06

**Authors:** Jenny Damcevska, Sima Dimitrijev, Daniel Haasmann, Philip Tanner

**Affiliations:** 1 Queensland Micro- and Nanotechnology Centre, Griffith University, Nathan, Brisbane, QLD 4111 Australia; 2 School of Engineering and Built Environment, Griffith University, Nathan, Brisbane, QLD 4111 Australia

**Keywords:** Engineering, Materials science

## Abstract

Due to superior material properties of SiC for high-voltage devices, SiC Schottky diodes are used in energy-conversion systems such as solar-cell inverters, battery chargers, and power modules for electric cars and unmanned aerial vehicles. The reliable operation of these systems requires the chip temperature of SiC Schottky diodes to be maintained within the limit set by the device package. This is especially crucial during surge-current events that dissipate heat within the device. As a thermal-management method, manufactures of commercial SiC Schottky diodes have introduced wafer thinning practices to reduce the thickness of the SiC chip and, consequently, to reduce its thermal resistance. However, this also leads to a reduction in the thermal capacitance. In this paper, we present experimental data and theoretical analysis to demonstrate that the reduced thermal capacitance has a much larger adverse effect in comparison to the beneficial reduction of the thermal resistance. An implication of the presented results is that, contrary to the adopted wafer thinning practices, SiC Schottky diodes fabricated without wafer thinning have superior surge-current capability.

## Introduction

Silicon carbide as a wide energy-gap semiconductor is revolutionising the next generation of power electronics. In addition to the wide energy gap, which results in ten times higher critical electric field in comparison to silicon, silicon carbide exhibits a high thermal conductivity and competitive electron mobility^1^. With several power-electronic devices benefiting from its superior characteristics, a pivotal development has been the SiC Schottky diode. Due to the beneficial features of modern SiC Schottky diodes, which includes fast switching speeds and recovery times, they are used in power-conversion systems such as photovoltaic solar inverters, power supplies, electric vehicles, battery chargers, and radio frequency applications.

A Schottky diode in its pure form is a metal–semiconductor junction that forms a rectifying energy barrier for the flow of electrons, and is referred to as a Schottky barrier diode (SBD). However, the dominant commercially available SiC Schottky diodes integrate p–n junctions in parallel with metal–semiconductor junctions, known as merged PN-Schottky (MPS) diodes. An additional feature of modern SiC Schottky diodes is the use of thinned down SiC chips, achieved by backgrinding SiC wafers from the standard 350 μm to as low as 110 μm^2–9^. A primary motivation for thinning the wafer is to reduce the electrical resistance of the chip^3,6,8,9^, which in turn reduces the thermal resistance and enables better heat removal^2,4,5,7^. The heat removal is important because the SiC chip temperature must be maintained within the specified operating limit, which is crucial for reliable operation during surge-current events. However, in addition to the thermal resistance, another fundamental parameter that impacts the SiC chip temperature is the thermal capacitance. As a result of wafer thinning practices, the thermal capacitance of the SiC chip is reduced, which has an opposing effect to the thermal resistance in terms of the SiC chip temperature during surge-current events.

The capability of Schottky diodes to withstand surge currents has been extensively reported in published literature, as it is regarded as one of the most significant reliability factors of power devices^10–19^. However, the association between wafer thinning and the surge-current capability of a Schottky diode is a novel field of research, with studies predominately reporting on the beneficial influence of wafer thinning as a way of enhancing the surge-current capability. This is consistent with the effect of reduced thermal resistance, however, there is limited experimental verification of the potential increase of SiC chip temperature due to the reduced thermal capacitance as a result of wafer thinning. Research has been focused on providing an in-depth assessment on the acceleration of heat removal due to the lower thermal resistance in devices with thinner chips^2,4,5^. There are also statements, including ones by the manufacturers of commercial SiC Schottky diodes, that wafer thinning in conjunction with the MPS structure enhances the surge-current capability of the device^5,7–9^. However, it has also been reported by manufacturers that there is a trade-off between decreasing the electrical resistance and decreasing the thermal capacitance^2^. Some simulation-based studies—with no experimental verification—demonstrate the ability to achieve higher surge currents with thinned SiC wafers, but only in conjunction with diffusion-solder die attach^8,9^. In the case where an experimental study was performed on the surge-current performance of a Schottky diode with a thinned SiC wafer, the tests were conducted on unpackaged devices^20^. However, the packaging is one of the primary aspects that influences the thermal properties of the SiC chip^21–23^. Therefore, there is no adequate quantitative analysis of the reduced thermal capacitance to show the impact of the commonly used wafer thinning on the temperature of the SiC chip of packaged Schottky diodes exposed to surge-current events.

In this paper, we show that wafer thinning causes an undesirable increase in the temperature of the SiC chip of packaged Schottky diodes. To understand the opposing effects of reduced thermal resistance and reduced thermal capacitance, an elementary thermal-circuit model is presented and verified by experimental data for both the Schottky barrier and commercial merged PN-Schottky diodes. The theoretical and experimental results presented in the paper provide a quantitative analysis of the frequently misinterpreted impact of wafer thinning on the SiC chip temperature during surge-current events.

## Chip temperature model

**Figure 1:**
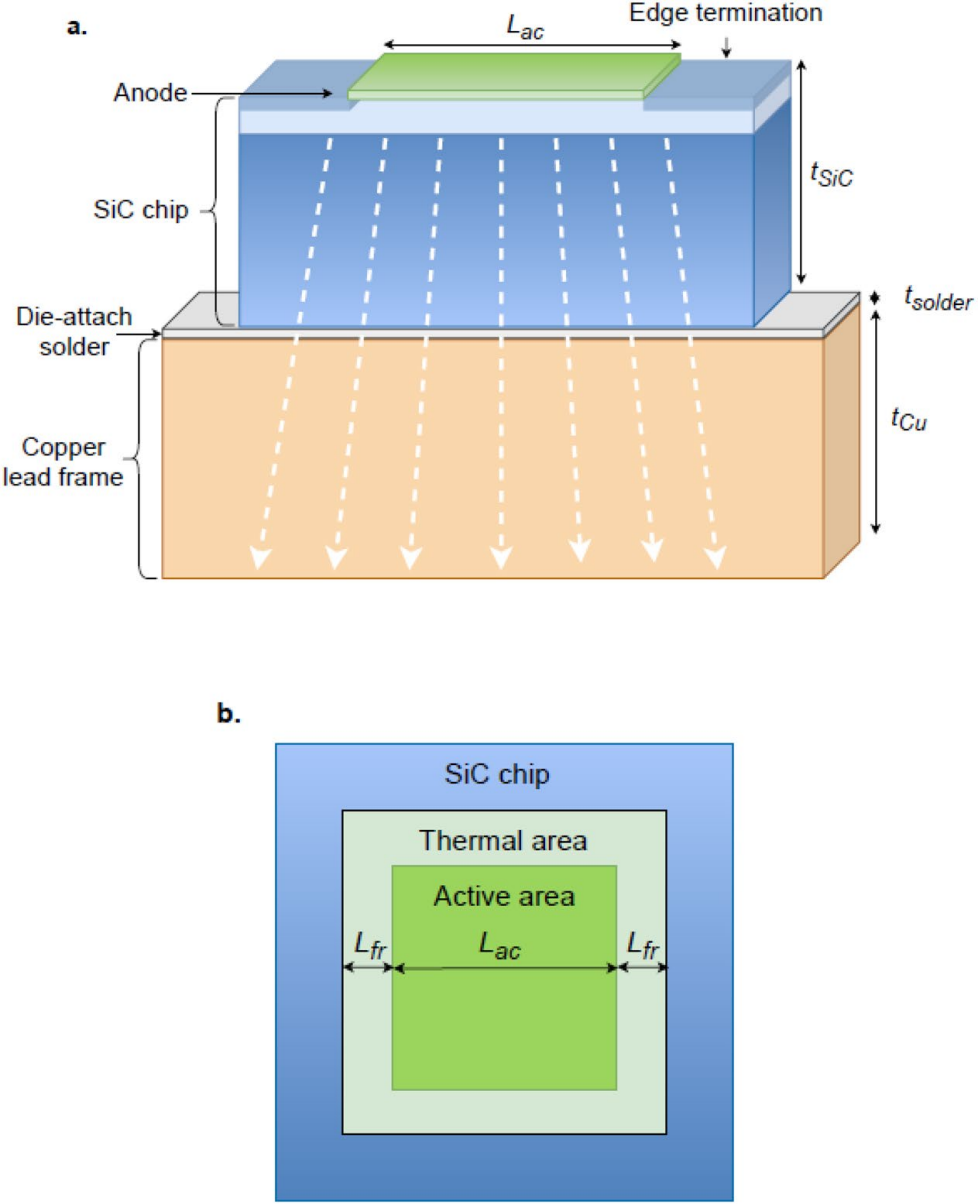
A cross-section and top view of a SiC Schottky barrier diode that illustrates (**a**) the material thicknesses (cross-section) and (**b**) the active and the thermal areas (top view).

The elementary model used to evaluate the thermal characteristics of a semiconductor device consists of a series connection of a thermal capacitance,${C}_{th}$, and a thermal resistance, ${R}_{th}$. To apply this model to the SiC Schottky diode, it is assumed that the temperature of the SiC chip depends on the absorption of dissipated energy by the thermal capacitance ${C}_{th}$ of the SiC chip, and on the rate of heat removal that depends on the thermal resistances of the SiC chip, die-attach solder, and the copper lead frame. The thermal capacitance of the SiC chip is^24^

1 $${C}_{th}={\rho }_{sic}{c}_{sic}{V}_{SiC}$$

where ${\rho }_{sic}$ is the mass density of 4H-SiC, ${c}_{sic}$ is the specific heat of 4H-SiC, and ${V}_{SiC}$ is the volume of the SiC chip. The thermal resistance of the SiC chip, the die-attach solder, and the copper lead frame is^24^

2 $${R}_{th}=\frac{{t}_{SiC}}{{k}_{SiC}{A}_{th}}+\frac{{t}_{solder}}{{k}_{solder}{A}_{th}}+\frac{{t}_{Cu}}{{k}_{Cu}{A}_{th}}=\frac{{R}_{th-sp}}{{A}_{th}}$$

where ${t}_{SiC}$, ${t}_{solder}$, and ${t}_{Cu}$ are the thicknesses of the SiC chip, the die-attach solder, and the copper, respectively, ${k}_{SiC}$, ${k}_{solder}$, and ${k}_{Cu}$ are the thermal conductivities of the SiC chip, the die-attach solder, and the copper, respectively, ${R}_{th-sp}$ is the specific thermal resistance, and ${A}_{th}$ is the thermal area. For the case of a square anode, the thermal area in Eq. (2) is

3 $${A}_{th}={\left({L}_{ac}+2{L}_{fr}\right)}^{2}$$

where ${L}_{ac}$ is the length of the electrically active region and ${L}_{fr}$ is the average length of the lateral flow of heat, as illustrated in Fig. 1. The average lateral length of heat flow is proportional to the material thickness, which is ${t}_{SiC}+{t}_{solder}+{t}_{Cu}$ for the case of thermal resistance and ${t}_{SiC}$ for the case of SiC thermal capacitance. Therefore, the volume of the thermal capacitance in Eq. (1) is

4 $$V={t}_{SiC}*{\left({L}_{ac}+2 {L}_{fc}\right)}^{2}$$

where ${L}_{fc}$ is the average length of the lateral flow of heat for the thermal capacitance, and is proportional to ${L}_{fr}$:

5 $${L}_{fc}/ {t}_{SiC}={L}_{fr} / ({t}_{SiC}+{t}_{solder}+{t}_{Cu})$$

Given that the thermal capacitance ${C}_{th}$ of the SiC chip absorbs the dissipated energy, the temperature inside the SiC chip, ${T}_{chip}$, is approximately uniform and equal to the junction temperature. This means that the temperature difference across the die-attach solder and the copper lead frame is ${T}_{chip}$—${T}_{case}$, where ${T}_{case}$ is the case temperature. As a result, the heat generated inside the SiC chip during a time interval $dt$ is^25^

6 $$d{Q}_{+}\left(t\right)={i}_{F}\left(t\right){v}_{F}\left(t\right)dt$$

where ${i}_{F}(t)$ and ${v}_{F}(t)$ are the forward current and the forward voltage, respectively, at time $t$. In turn, the heat removed from the SiC chip during a time interval $dt$ is due to the temperature difference acting as the driving force for the flow of heat and the thermal resistance opposing it, which is^26^

7 $$d{Q}_{-}(t)=\frac{{T}_{chip}-{T}_{case}}{{R}_{th}}dt$$

The applicability of this equation will be verified by experimental data; however, it is justified by the fact that the thermal resistance of 4H-SiC is much lower than the series thermal resistance of the die-attach solder and the copper lead frame.

The change in chip temperature during a time interval $dt$ is proportional to the difference of heat that is generated in the chip and removed from the chip, ${dQ}_{+}-{dQ}_{-}$, and inversely proportional to the thermal capacitance, ${C}_{th}$^27^. Therefore,

8 $${dT}_{chip}=\frac{{dQ}_{+}-{dQ}_{-}}{{C}_{th}}=\frac{{i}_{F}\left(t\right){v}_{F}\left(t\right)dt-\left[\left({T}_{chip}-{T}_{case}\right)/{R}_{th}\right]dt}{{C}_{th}}$$

Equation (8) can be rearranged in the form of a linear first-order differential equation:

9 $$\frac{d{T}_{chip}}{dt}+ \frac{1}{{R}_{th}{C}_{th}}{T}_{chip}=\frac{{i}_{F}{v}_{F}}{{C}_{th}}+\frac{{T}_{case}}{{R}_{th}{C}_{th}}$$

The final equation for the chip temperature is obtained as the solution of Eq. (9):

10 $${T}_{chip}=\mathrm{exp}\left(-\frac{t}{{R}_{th}{C}_{th}}\right)\left[{T}_{case}+{\int }_{0}^{t}g\left(t\right)\mathrm{exp}\left(\frac{t}{{R}_{th}{C}_{th}}\right)dt\right]$$

11 $$\mathrm{where }\;g\left(t\right)=\frac{{i}_{F}{v}_{F}}{{C}_{th}}+\frac{{T}_{case}}{{R}_{th}{C}_{th}}$$

This chip-temperature model, given by Eqs. (10) and (11), is verified by experimental data in the following section.

## Experimental verification

The experimental chip-temperature data was derived from the measured isothermal forward-bias current–voltage characteristics and measured ${i}_{F}$ and ${v}_{F}$ data during 10-ms half-sinewave surge current pulses, as defined in the methodology section. SBD’s with a chip thickness of 350 μm and MPS diodes with a chip thickness of 110 μm were used to ensure that the model was evaluated for different chip thicknesses. In addition, measurements were performed on diodes with three DC blocking voltages, including 650 V, 1200 V, and 1700 V for the SBD, and 1200 V for the MPS diode, with each voltage range tested with three peak surge currents.

**Figure 2:**
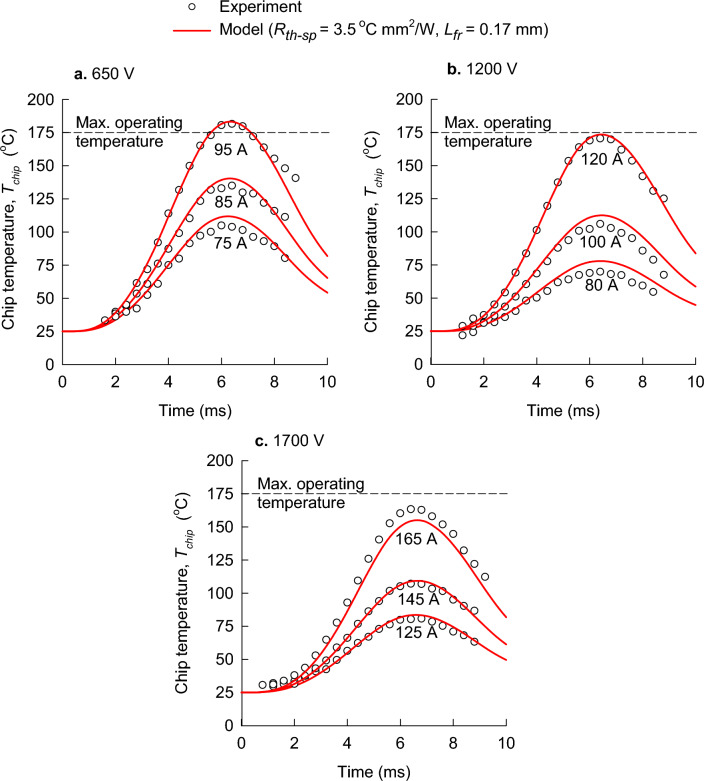
Comparison of experimental data for Schottky barrier diodes (symbols) and the theoretical chip-temperature model (lines) for three peak surge currents per blocking voltage: (**a**) 650 V, (**b**) 1200 V, and (**c**) 1700 V.

The theoretical and experimental results for the SBD’s are illustrated in Fig. 2. Two parameters, the specific thermal resistance, ${R}_{sp-th}$, and the lateral length of heat flow, ${L}_{fr}$, were used for all nine data sets, which includes the three DC blocking voltages and three peak surge currents. Good agreement was achieved between the theoretical chip-temperature model and the experimental data with single values for the specific thermal resistance, ${R}_{sp-th}$, and the length of the lateral flow of heat, ${L}_{fr}$.

Furthermore, the value of 3.5 °C ${\mathrm{mm}}^{2}/\mathrm{W}$ for the specific thermal resistance is consistent with the typical material thicknesses and parameters, as shown in Table 1. The value of the lateral flow of heat is also consistent with the typical material thicknesses.

**Table 1:** Specific thermal resistances of 4H-SiC, die-attach solder, and copper lead frame, calculated from the typical thickness of each layer and the values for the thermal conductivity.

	Thickness $(\mathrm{mm})$	Thermal Conductivity $k$ ($\mathrm{W}/\mathrm{ mm}\, ^\circ \mathrm{C}$)	Specific thermal resistance ${R}_{sp-th}$ ($^\circ \mathrm{C}{\, \mathrm{mm}}^{2}/\mathrm{W}$)
4H-SiC	0.350	0.490	0.7
Die-attach solder	0.030	0.036	0.8
Copper lead frame	0.800	0.394	2.0
Total			3.5

**Figure 3:**
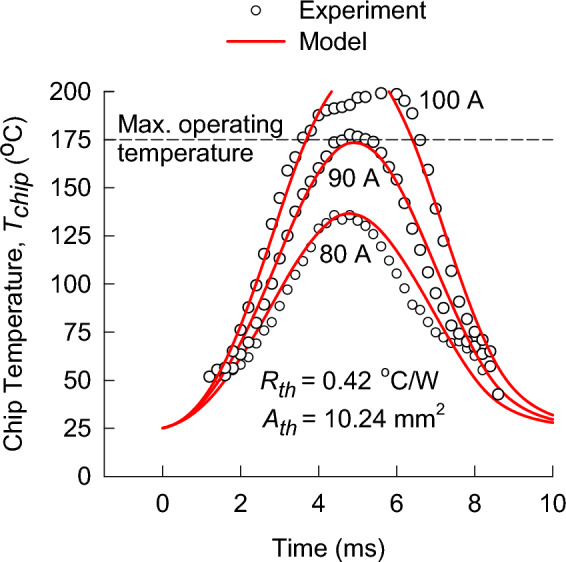
The experimental data (symbols) and the theoretical-chip temperature model (lines) for three peak surge currents applied to a commercial 1200 V MPS diode.

The results for the commercial 1200 V MPS diode are illustrated in Fig. 3. The theoretical-model results were obtained using the thermal area, ${A}_{th}$, as the single fitting parameter. The value used for the thermal resistance, ${R}_{th}$, was within the relevant datasheet specifications. The values of these two parameters were used for all three surge currents. The achieved good agreement up to the chip temperature of $175 ^\circ \mathrm{C}$ demonstrates that the theoretical model is applicable to the MPS diode with a thinned wafer of 110 μm.

In this paper, we focus the considerations on chip temperatures up to $175 ^\circ \mathrm{C}$ because this is the maximum rated chip temperature in all available commercial diodes^22^. In other words, the chip temperature must not exceed $175 ^\circ \mathrm{C}$ to ensure reliable operation of the device. A small deviation of the experimental data from the theoretical curve above $175 ^\circ \mathrm{C}$ can be observed in Fig. 3 for the highest peak current of $100\; \mathrm{A}$. We conclude that this is due to the internal structure of the MPS diode that changes the operating condition from a unipolar state of conduction to a bipolar state. Consequently, the relationship between the measured ${i}_{F}$ and ${v}_{F}$ data during the surge-current pulse and the isothermal current–voltage characteristics is distorted.

## Analysis of the impact of wafer thinning on surge-current capability

**Figure 4:**
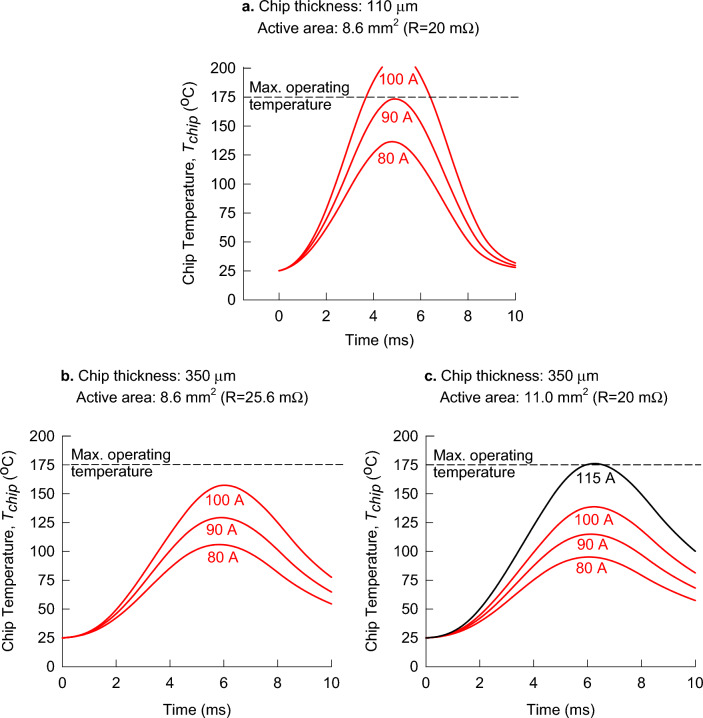
Chip temperatures of 1200 V MPS diodes in response to different peak surge currents: (**a**) the commercially available 1200 V MPS diode with a wafer thickness of 110 μm, as shown in Fig. 3; (**b**) 1200 V MPS diode with the chip thickness of 350 μm and the same area as the commercial diode; (**c**) 1200 V MPS diode with the chip thickness of 350 μm and increased area to match the electrical resistance of the commercial diode.

The modelling results for the chip temperature of the commercial 1200 V MPS diode in response to three peak surge currents are reproduced in Fig. 4a. The measured electrical resistance of the SiC chip, which is in series with the Schottky barrier and PN diodes, is ${R}_{(a)}=20\; \mathrm{m\Omega }$. Using the value of lateral length of heat flow that corresponds to ${t}_{SiC (a)}=110\; \mathrm{\mu m}$, which is ${L}_{fr}=0.135\; \mathrm{mm}$, we can estimate the active anode area:

12 $$\begin{aligned} A_{ac \left( a \right)} = & \left( {\sqrt {A_{th \left( a \right)} } - 2L_{fr \left( a \right)} } \right)^{2} \\ = & \left( {\sqrt {10.24} - 2 \times 0.135} \right)^{2} = 8.6 \;{\text{mm}}^{2} \\ \end{aligned}$$

Using the same active anode area for a diode without wafer thinning (${t}_{SiC (b)}=350\; \mathrm{\mu m}$) in the model for chip temperature, we obtain the chip temperature results shown in Fig. 4b. Note that the lateral length of heat flow in this case, ${L}_{fr (b)}=0.17 \;\mathrm{mm}$, corresponds to ${t}_{SiC (b)}=350\; \mathrm{\mu m}$. The results show that the chip temperatures are much lower for the same peak surge currents, meaning that this diode exhibits superior surge-current capabilities without wafer thinning.

The increase in chip thickness from 110 to 350 μm does increase the thermal resistance; however, it also increases the thermal capacitance, and this result shows that the impact of thermal capacitance is dominant. This is illustrated in Table 2 by the values of specific thermal resistances (${R}_{sp-th}$) and thermal capacitances per unit area (${C{\prime}}_{th}$) for each diode. Specifically, the numbers in Table 2 show that the wafer thinning from 350 to 110 μm reduces the thermal capacitance per unit area by 3.2 times, which is a much larger adverse factor in comparison to the beneficial reduction of specific thermal resistance by only 1.2 times.

**Table 2:** Specific thermal resistances and thermal capacitances per unit area, illustrating that the decreased chip thickness from 350 to 110 μm has a minimal beneficial impact on the specific thermal resistance, whereas the adverse decrease of the thermal capacitance per unit area is proportional to the decreased chip thickness.

Chip thickness (μm)	Components of specific thermal resistance,${{\varvec{R}}}_{{\varvec{s}}{\varvec{p}}-{\varvec{t}}{\varvec{h}}}$	Total ${{\varvec{R}}}_{{\varvec{s}}{\varvec{p}}-{\varvec{t}}{\varvec{h}}}$ (${^\circ \mathrm{C}\, \mathbf{m}\mathbf{m}}^{2}/\mathbf{W}$)
110	${R}_{sp-th (a)}=\frac{{t}_{SiC (a)}}{{k}_{SiC}}+\frac{{t}_{solder}}{{k}_{solder}}+\frac{{t}_{Cu}}{{k}_{Cu}}=0.22+0.83+2.03$	3.1
350	${R}_{sp-th (b)}=\frac{{t}_{SiC (b)}}{{k}_{SiC}}+\frac{{t}_{solder}}{{k}_{solder}}+\frac{{t}_{Cu}}{{k}_{Cu}}=0.71+0.83+2.03$	3.6

Chip thickness (μm)	Thermal capacitance per unit area,${C{\prime}}_{th}$	Total ${C{\prime}}_{th}$ ($\mathrm{J}/^\circ \mathrm{C}\, {\mathrm{m}}^{2}$*)*
110	${{C}{\prime}}_{th \left(a\right)}= {\rho }_{SiC}{c}_{SiC}{t}_{SiC \left(a\right)}=3210\times 700\times 110\times {10}^{-6}$	$247.2$
350	${C{\prime}}_{th (b)}= {\rho }_{SiC}{c}_{SiC}{t}_{SiC (b)}=3210\times 700\times 350\times {10}^{-6}$	$786.45$

As mentioned previously, the main motivation for wafer thinning is to reduce the specific electrical resistance, which also reduces the thermal resistance to enable better heat removal. Therefore, it is useful to analyse the impact of an increased diode area that is needed to achieve the same electrical resistance without wafer thinning. As shown in Fig. 4b, the electrical resistance of the diode with the same active anode area and the thicker chip is ${R}_{(b)}=25.6 \;\mathrm{m\Omega }$. This is due the increase of specific electrical resistance from $\rho \cdot{t}_{SiC \;\left(a\right)}$ to $\rho \cdot {t}_{SiC\; \left(b\right)}$, where $\rho =2\times {10}^{-4} \;\mathrm{\Omega m}$ is the resistivity of the SiC wafer:

13 $${R}_{(b)}={R}_{(a)}+\rho \left({t}_{SiC\; (b)}-{t}_{SiC\; (a)}\right)/{A}_{ac \;(a)}=25.6 \;\mathrm{m\Omega }$$

To match the electrical resistance of the thinned diode at ${R}_{(a)}=20 \;\mathrm{m\Omega }$, the active anode area of the diode without thinning should be increased to:

14 $${A}_{ac\; (c)}={A}_{ac\; (b)}\left({R}_{(b)}/{R}_{(a)}\right)=11.0\; {\mathrm{mm}}^{2}$$

This increase in diode area has a beneficial effect on the surge-current capability by both an increase in the thermal capacitance and a reduction in the thermal resistance. Figure 4c shows that the chip temperature for the peak surge current of 100 A drops to around $140 ^\circ \mathrm{C}$. This value is well below the peak temperature of the thinned diode with the same electrical resistance (Fig. 4a), which exceeded the standardised limit of $175 ^\circ \mathrm{C}$. Table 3 summarises the results for the chip temperatures of the analysed MPS diodes. The results show that the temperature limit of $175 ^\circ \mathrm{C}$ corresponds to the peak surge current of 115 A in the case of a non-thinned diode with increased area, which is a 28% improvement in comparison to the peak surge current of around 90 A in the case of the thinned diode with the same electrical resistance.

**Table 3:** Comparison of chip temperatures for different peak surge currents, illustrating the impact of increased chip thickness and increased active area of the MPS diode.

Peak surge-current (A)	Chip temperature (°C)		
	${t}_{SiC}=110 \;\mathrm{\mu m}$ ${A}_{ac}=8.6\;\mathrm{ m}{\mathrm{m}}^{2}$ $\left(R=20.0\;\mathrm{ m\Omega }\right)$	${t}_{SiC}=350\;\mathrm{ \mu m}$ ${A}_{ac}=8.6\;\mathrm{ m}{\mathrm{m}}^{2}$ $\left(R=25.6 \;\mathrm{m\Omega }\right)$	${t}_{SiC}=350 \;\mathrm{\mu m}$ ${A}_{ac}=11.0\; {\mathrm{mm}}^{2}$ $\left(R=20.0\; \mathrm{m\Omega }\right)$
80	136.5	106	95
90	173	129	115
100	211	157	139
115			175

As previously mentioned, it has been reported that a SiC MPS diode with a chip thickness of 110 μm was able to withstand higher surge currents than with a chip thickness of 350 μm, due to the addition of diffusion soldering die attach. The MPS diode used in this study was also fabricated with diffusion soldering, however, the same surge currents applied to the SBD with no diffusion soldering result in lower chip temperatures, as can be seen by comparing Figs. 2b and 3. On the other hand, the redesigned MPS diode with a chip thickness of 350 μm and a larger active area (Fig. 4c) has the same chip thickness, similar active area, and similar surge-current capability as the 1200 V SBD (Fig. 2b). This shows that the chip thickness and active area are the dominant factors for the surge-current capability, irrespective of the internal diode structure (SBD or MPS diode).

The impact of reduced thermal resistance by the diffusion soldering can be estimated by comparing the specific thermal resistances shown in Table 2. Assuming that the diffusion soldering eliminates the thermal resistance of the solder, the total specific resistance of a diode with its chip thinned down to 110 μm is ${2.3 ^\circ \mathrm{C}\,\cdot \mathrm{mm}}^{2}/\mathrm{W}$. This specific resistance is only 1.35 times smaller in comparison to a diode with the chip thickness of 110 μm and standard solder. In comparison to a diode with a chip thickness of 350 μm and standard solder, the specific thermal resistance of a diode combining wafer thinning and diffusion soldering is 1.6 times smaller. However, these favourable reductions of the specific thermal resistance are still smaller in comparison to the adverse effect of 3.2 times smaller thermal capacitance per unit area due to the wafer thinning from 350 to 110 μm. This analysis shows that the copper lead frame acts as the dominant thermal resistance to the heat flow, whereas the SiC chip acts as the dominant thermal capacitance whose volume determines how much heat can be absorbed at a given chip temperature.

With a primary incentive of reducing the electrical and thermal resistance of the Schottky diode by wafer thinning, there is also a cost incentive due to the reduced SiC chip area. However, additional factors need to be taken into consideration that increase the cost, such as yield loss as a result of wafer breakage during backgrinding. The presented analysis identifies the thermal capacitance as the dominant parameter that directly influences the ability of the device to absorb thermal energy during surge current events, which directly impacts the temperature of the SiC chip. Therefore, the superior surge-current capability of diodes fabricated with non-thinned wafers and larger areas can justify the potential increase in fabrication cost.

## Methodology

### Device fabrication

**Figure 5:**
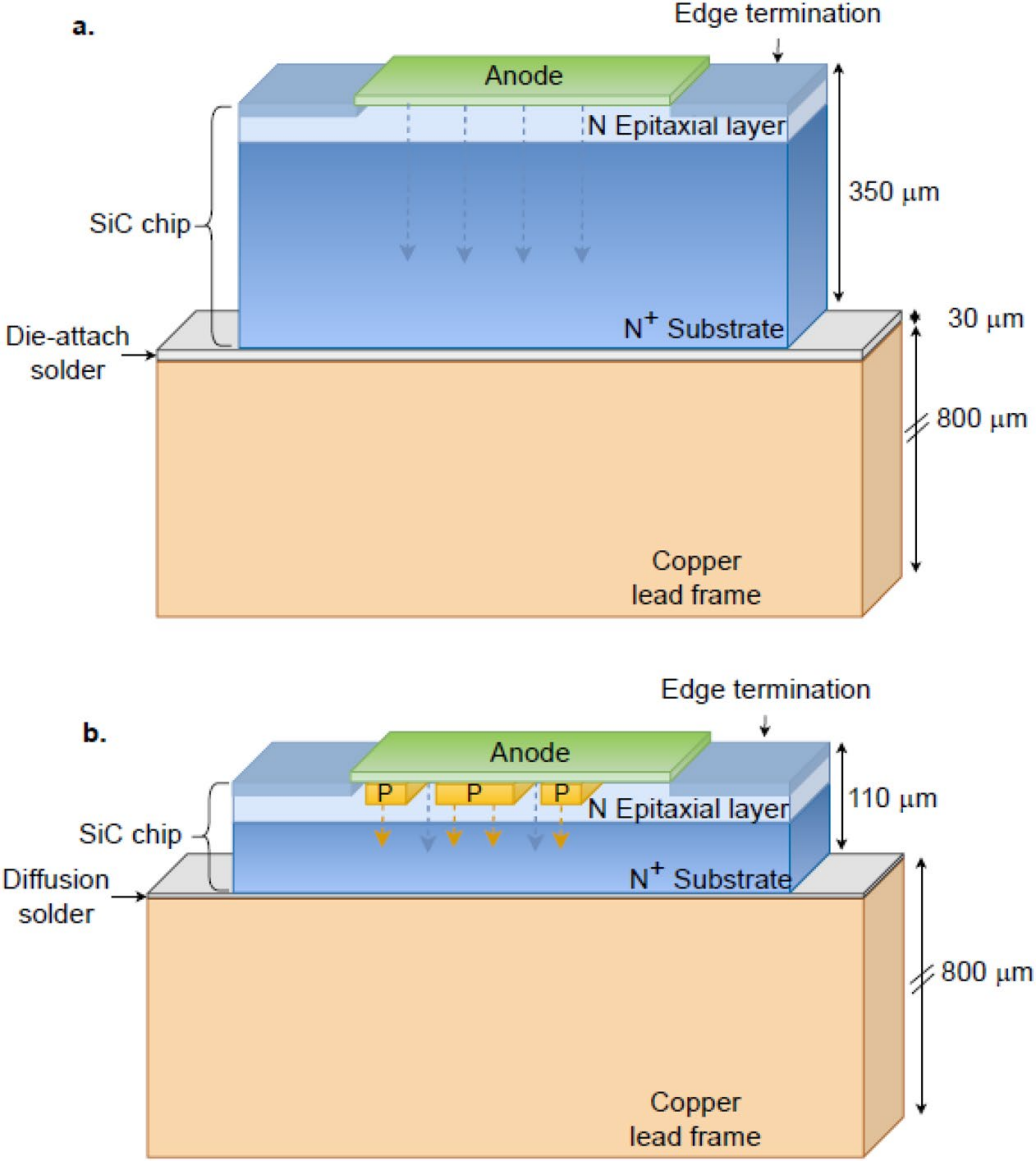
Illustrative cross sections of (**a**) Schottky barrier diode (SBD) and (**b**) merged PN-Schottky (MPS) diode.

Figure 5 illustrates the cross-sections of two types of SiC Schottky diodes used for the experimental and theoretical analyses in this paper: (a) Schottky barrier diode (SBD) and (b) merged PN junctions with Schottky diodes (MPS).

Schottky barrier diodes designed for three blocking voltages (650 V, 1200 V, and 1700 V) were fabricated using 150-mm 4H-SiC wafers with the thickness of 350 μm and resistivity of $2\times {10}^{-4} \;\mathrm{\Omega m}$.

The Schottky barriers on the N-type drift regions were formed by sputtering 100-nm thick titanium and 4-μm thick aluminium. In addition to a suitable edge termination, the areas outside the anode were passivated by deposited SiO_2_. The Ohmic contact on the back of the wafer was formed by sputtering 100-nm thick nickel and 2-μm thick silver. The diodes were packaged in TO247 standard packages with the standard die-attach solder. These diodes exhibited the forward- and reverse-bias current–voltage characteristics according to the fundamental current mechanisms described in the paper by Nicholls et al.^28^. The commercial 1200 V MPS diode used in the paper was fabricated on a 4H-SiC wafer thinned down to 110 μm. The diode package was TO247, with diffusion soldering of the SiC chip to copper frame.

### Measurements and analysis

#### Isothermal measurements of current–voltage characteristics

**Figure 6:**
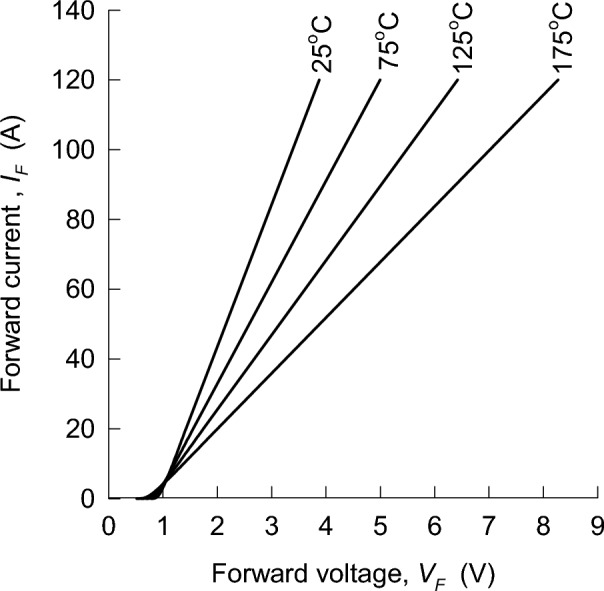
An example of isothermal current–voltage characteristics for a 1200 V Schottky-barrier diode, as they are usually shown in datasheets.

The isothermal current–voltage characteristics for the Schottky barrier diode were performed on a probe station from Materials Development Corporation (MDC). Each device under test was heated on a hot chuck to four predefined temperatures, from 25 °C to 175 °C, using a Quiet Chuck DC Controller that was connected to the MDC system. At each set temperature, the forward current–voltage characteristics were measured using an Agilent B1505A Power Device Analyzer and Curve Tracer from Agilent Technologies, with a pulse duration of 380 μs taken every 100 ms to ensure the junction was not heated. The very short pulses of 380 μs were imperative to the isothermal measurements to ensure the junction temperature was the same as the chuck temperature. Typical isothermal current–voltage characteristics for the Schottky barrier diode are shown in Fig. 6. The isothermal current–voltage characteristics for the merged PN-Schottky diode were extracted from the relevant commercial datasheet.

##### Surge current measurements

**Figure 7:**
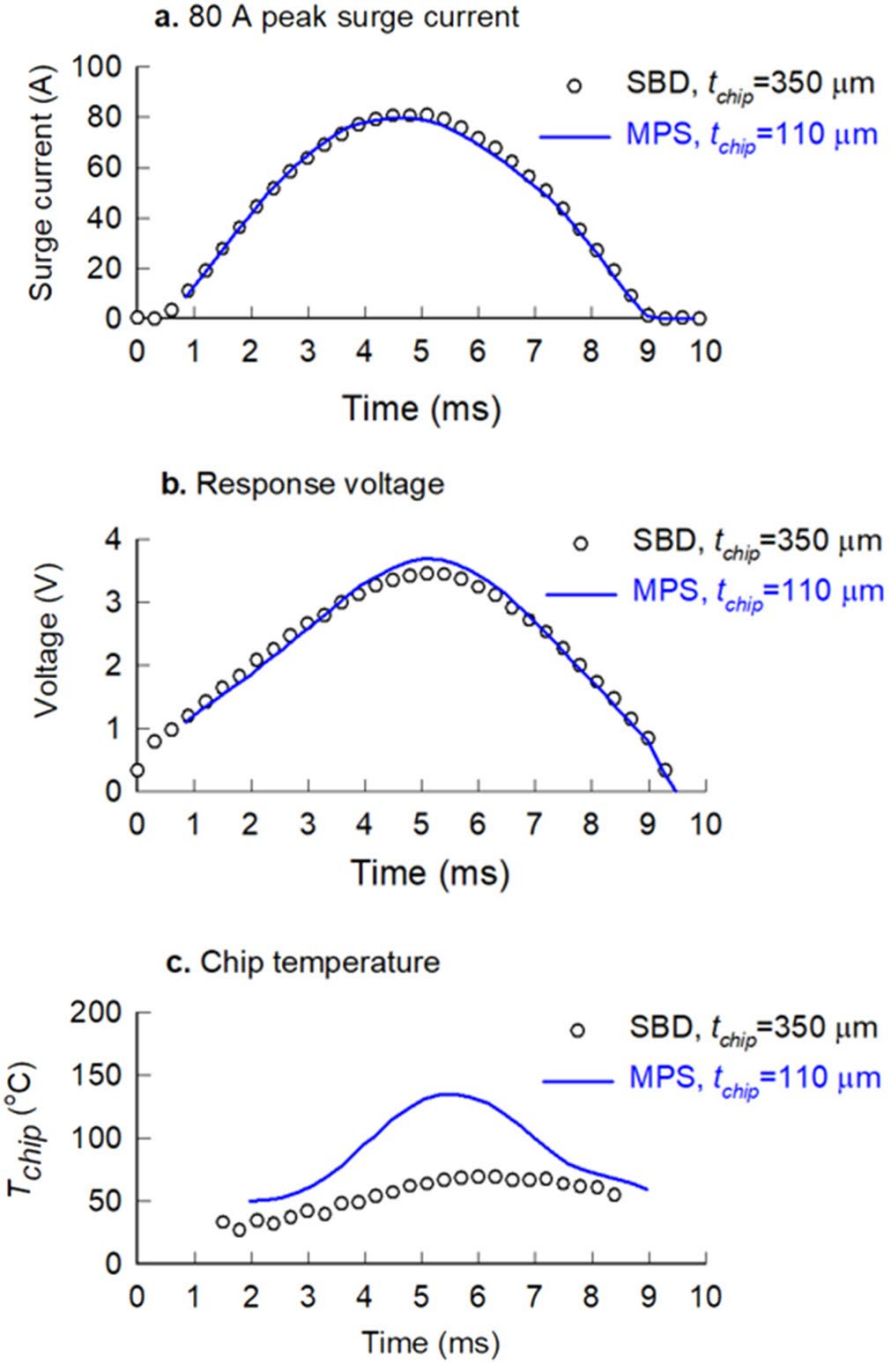
Typical surge-current measurements: (**a**) a 10-ms half-sinewave with a peak surge current of 80 A, (**b**) the response voltage, and (**c**) the chip temperature for Schottky barrier diode with a wafer thickness of 350 μm (symbols) and the merged PN-Schottky diode with a wafer thickness of 110 μm (lines).

The surge-current measurements were conducted using a 50 Hz sinusoidal current source for both the Schottky barrier and merged PN-Schottky diodes. Each device under test was connected with a metal oxide thermal paste between the metallisation of the package and the heat sink, and then pulsed with a 10-ms half-sinewave. The set of peak forward surge currents for both the Schottky barrier and the merged PN-Schottky diodes for each DC blocking voltage are illustrated in Table 4. The current and voltage values were recorded simultaneously for each pulse using a PicoScope 2000 series dual channel oscilloscope from Pico Technology. Typical half-sinewave surge-current pulses and the responding voltages are shown in Fig. 7a and b, respectively.

**Table 4:** Peak forward surge currents for the Schottky barrier and merged PN-Schottky diodes for all three DC blocking voltages.

Blocking voltage (V)	Peak forward surge currents (A)	
	Schottky barrier diode (SBD)	Merged PN-Schottky diode (MPS)
650	75 85 95	
1200	80 100 120	80 90 100
1700	125 145 165

**Figure 8:**
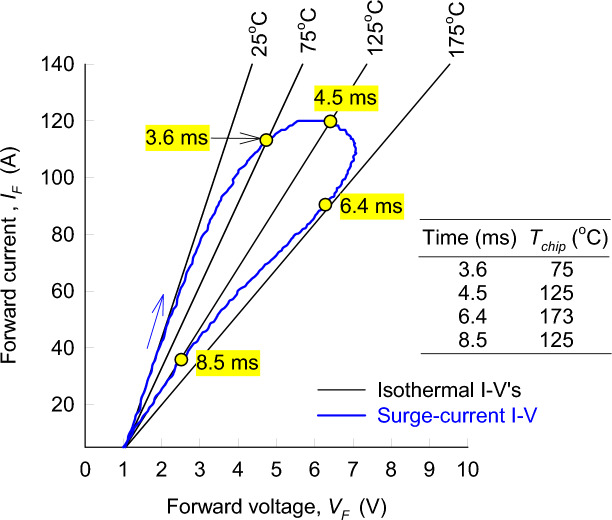
Illustration of the time-dependent chip-temperature measurement based on isothermal current–voltage (I–V) characteristics, using the example of a 1200 V Schottky barrier diode and 120 A peak surge current.

The chip temperature during the surge-current events was determined from the measured surge current and the response voltage by matching their values to isothermal measurements at different temperatures, and non-linear data interpolation between the measured temperatures. The measured surge current and the response voltage are shown by the blue loop in Fig. 8. The loop shows the effect of chip heating when the surge current rises towards the peak, which results in a higher voltage for the same current when the current drops below the peak. The isothermal current–voltage characteristics for this diode are also shown in Fig. 8 by the black lines. At 3.6 ms, the surge current and the responding voltage match the isothermal current and voltage at 75 °C, which means that the chip temperature is equal to 75 °C at 3.6 ms. The temperature rises to 125 °C at 4.5 ms, reaches its maximum of around 173 °C at 6.4 ms, and then decreases back to 125 °C at 8.5 ms. This describes the method of time-dependent chip-temperature measurement with a note that non-linear interpolation is used to obtain a continuous chip temperature during the surge cycle. An example of a continuous chip temperature during a surge-current cycle is shown in Fig. 7c.

An important observation regarding Fig. 7c is the measured difference between the chip temperature of the SBD and the MPS diode. Figure 7a and b show that the power dissipation in both diodes is similar. For diodes with equal chip thicknesses, this would mean that the chip temperature is the same. However, in this case the reduced wafer thickness of the MPS diode results in a significant increase in the chip temperature due to the reduction in the thermal capacitance. It happens that the response voltage of the MPS diode with higher chip temperatures is similar to the SBD diode, but that is because the MPS diode changes its operating conditions from unipolar to bipolar state of conduction, which reduces the forward voltage.

## Conclusion

In this paper, we present experimental data and theoretical analysis of the surge-current capability of SiC Schottky diodes, with a focus on the effects of reduced thermal capacitance and reduced thermal resistance resulting from the commonly adopted industry practice of wafer thinning. The key result is that the wafer thinning is unfavourable because the reduced thermal capacitance has a greater adverse effect in comparison to the beneficial reduction of the thermal resistance. Experimental data shows that a representative commercial 1200 V diode with an MPS structure and a wafer thickness of 110 μm reaches the chip temperature around the set maximum of 175 °C during the peak surge current of 90 A. In contrast, a 1200 V SBD with a wafer thickness of 350 μm reaches the chip temperature of approximately 175 °C at peak surge currents of 120 A, which is 30 A greater than the diode with a thinner SiC chip. Further analysis of the impact of wafer thinning shows that the specific thermal resistance of the MPS diode without wafer thinning is only 1.16 times higher. On the other hand, the thermal capacitance of the MPS diode without wafer thinning is 3.18 times higher, illustrating that the thermal capacitance is dominant. The analysis in the paper also includes the effect of chip area, given that a reduction in the specific electrical resistance by wafer thinning also results in a smaller chip area. The analysis shows that an area increase of the MPS diode with a chip thickness of 350 μm, needed to match the electrical resistance of the MPS diode with a chip thickness of 110 μm, results in both a favourable reduction in the thermal resistance and a favourable increase in the thermal capacitance. This MPS diode has a similar area, the same chip thickness, and similar surge-current capability as the comparable SBD diode, which shows that the chip thickness and chip area rather than the MPS structure determines the peak surge current that maintains the chip temperature within the set limit of 175 °C.

## Data Availability

The datasets that were analysed and support the findings of this study are available on request from the corresponding author.
